# Chlorine, Bromine, and Iodine as Disinfectants

**Published:** 1885-04

**Authors:** 


### Chlorine, Bromine, and Iodine as Disinfectants.

Dr. Geo. H. Rohe thus sums up, in the *Medical News*, the disinfecting qualities of the above named agents:

1st. Chlorine is an efficient disinfectant when present in the proportion of one part in 100; provided the air and the objects to be disinfected are in a moist state, and the exposure continues for upwards of one hour.

2d. Chlorine, when used in sufficient concentration to act as a trustworthy disinfectant, injures colored fabrics and wearing apparel.

3d. Bromine is also an efficient disinfectant in the proportion of 1 part in 500; provided the air be in a moist state, and the exposure continues for upwards of three hours.

4th. Iodine, in solution, is an efficient disinfectant in the proportion of 1 part in 500; the exposure continuing for two hours.

5th. The use of chlorine, and in a greater degree of Bromine, requires considerable experience in management; when carelessly handled, they may cause inconvenient, or even dangerous, symptoms in persons using them; for these reasons they are not suitable as disinfectants for popular use.

